# Review of Canadian species of the genera *Gnathusa* Fenyes, *Mniusa* Mulsant & Rey and *Ocyusa* Kraatz (Coleoptera, Staphylinidae, Aleocharinae)

**DOI:** 10.3897/zookeys.412.7282

**Published:** 2014-05-29

**Authors:** Jan Klimaszewski, Reginald P. Webster, David W. Langor, Caroline Bourdon, H.E. James Hammond, Greg R. Pohl, Benoit Godin

**Affiliations:** 1 Natural Resources Canada, Canadian Forest Service, Laurentian Forestry Centre, 1055 du P.E.P.S., P.O. Box 10380, Stn. Sainte-Foy, Quebec, Quebec, Canada G1V 4C7; 2 24 Mill Stream Dr., Charters Settlement, New Brunswick, Canada E3C 1X1; 3 Natural Resources Canada, Canadian Forest Service, Northern Forestry Centre, 5320-122 Street, Edmonton, Alberta, Canada T6H 3S5; 4 14A Thompson Rd., Whitehorse, Yukon, Canada Y1A 0C4

**Keywords:** Staphylinidae, *Gnathusa*, *Mniusa*, *Ocyusa*, Taxonomy, Canada

## Abstract

Four species of *Gnathusa* Fenyes (*G. alfacaribou* Klimaszewski & Langor, *G. caribou* Lohse, *G. eva* Fenyes, and *G. tenuicornis* Fenyes) occur in the Nearctic and in Canada. Three species of *Ocyusa* Kraatz (*O. asperula* Casey, *O. californica* Bernhauer, *O. canadensis* Lohse), and three species of *Mniusa* Mulsant and Ray (*M. minutissima* (Klimaszewski & Langor), *M. yukonensis* (Klimaszewski & Godin), and *M. odelli* Klimaszewski & Webster, **sp. n.**), are known from the Nearctic and all but *O. californica* occur in Canada. The recently described *Gnathusa minutissima* Klimaszewski and Langor and *Ocyusa yukonensis* Klimaszewski and Godin, are transferred here to the genus *Mniusa* Mulsant & Rey. New provincial and state records are reported for: *G. eva* (Alberta), *G. tenuicornis* (Alberta, Oregon, and New Brunswick), *O. canadensis* (New Brunswick and Newfoundland), *M. minutissima* (New Brunswick), and *M. yukonensis* (Nova Scotia, New Brunswick, Quebec, and British Columbia). The female of *M. yukonensis* was discovered and is illustrated for the first time. The genus *Mniusa* is reported for the first time from Canada and represents the first confirmed generic record for North America. Keys for identification of all Canadian species, images of body and genital structures, maps showing distribution mainly in Canada, and new bionomics data are provided.

## Introduction

Fenyes (1909) described the genus *Gnathusa* and two species from the Nearctic region, *Gnathusa eva* from California and *Gnathusa tenuicornis* from British Columbia. More recently, Lohse (Lohse et al. 1990) described a northern species, *Gnathusa caribou* from Canada (YT, NWT) and Alaska, and later Klimaszewski and Langor (Klimaszewski et al. 2011) described an additional species, *Gnathusa alfacaribou*, from Newfoundland and Labrador, a species closely related to *Gnathusa caribou*. Recently, more specimens of *Gnathusa* have become available for study, resulting in new range extensions but no additional new species. It appears that species of this genus are confined to the Rocky Mountains and northern Canada.

*Gnathusa* is sometimes confused in North American collections with some species of the genus *Ocyusa* Kraatz (1856). *Ocyusa* was originally described in Europe and currently includes nine species, excluding two species of *Mniusa* Mulsant & Rey (Smetana 2004, in Löbl and Smetana 2004). However, Palm (1972) and Ashe (2000) considered *Mniusa* to be a subgenus of *Ocyusa*. Osswald et al. (2013) proposed a molecular phylogeny of the rove beetle tribe Oxypodini where they recognised *Mniusa* and *Ocyusa* as two distinct genera. They classified *Mniusa* as closely related to *Gnathusa*, and *Ocyusa* as closely related to the *Oxypoda*, *Devia*, *Ilyobates*, *Tetraleucopora* and *Ocalea* group of genera. Assing (1998) revised species of Palaearctic *Zoosetha* Mulsant & Rey and provided a taxonomic history of related genera including *Ocyusa*. *Ocyusa asperula* was the first species of this genus described in the Nearctic region, from Rhode Island (Casey 1894). It was later reported by Bernhauer (1906) from Iowa and Massachusetts under the synonymic name *Ocyusa brevipennis*. Webster et al. (2009) reported it for the first time from Canada in New Brunswick. Bernhauer (1906) described *Ocyusa californica* from California, and this species is not found in Canada. Lohse (Lohse et al. 1990) described a new northern species, *Ocyusa canadensis* from Yukon Territory and Alaska. Brunke et al. (2012) reported this species from Ontario, and we report it here from Newfoundland and Labrador, and New Brunswick. Klimaszewski et al. (2011, 2012) described *Gnathusa minutissima* from the province of Newfoundland and Labrador, and *Ocyusa yukonensis* from Yukon Territory, but they are here transferred to the genus *Mniusa* on the basis of pronotal pubescence along midline directed anteriad in apical third of median line of disc, absence of fronto-clypeal suture, L-shaped spermathecal neck connected to thin stem, and apical margin of male tergite 8 sinuate laterally and produced medially. *Mniusa minutissima* is newly recorded from New Brunswick. Recently, *Mniusa yukonensis* was described from the Yukon Territory under the genus *Ocyusa* (Klimaszewski et al. 2012), and we now provide new records of this species from British Columbia, Quebec, Nova Scotia, and New Brunswick. Here, we have discovered and described another *Mniusa* species, *Mniusa odelli*, which has similar genitalic features to those of *Mniusa yukonensis*, but has a different body form. To facilitate identification of species of *Gnathusa*, *Mniusa*, and *Ocyusa* in Canada, we review their diagnostic features, and provide keys to identification. We also provide extensive illustrations of diagnostic characters, including external body images and genital structures.

## Materials and methods

Over 140 adults of the genus *Gnathusa* and 100 adults of *Ocyusa* and *Mniusa* from Canada and the United States were studied, and most specimens were dissected to examine the genital structures and in some cases, mouthpart structures. The genital structures were dehydrated in absolute alcohol, mounted in Canada balsam on celluloid microslides, and pinned with the specimens from where they originated. Images of the entire body and the genital structures were taken using an image processing system (Nikon SMZ 1500 stereoscopic microscope; Nikon Digital Camera DXM 1200F, and Adobe Photoshop software).

Morphological terminology mainly follows that used by Seevers (1978) and Klimaszewski et al. (2011). The ventral side of the median lobe of the aedeagus is considered to be the side of the bulbus containing the foramen mediale, the entrance of the ductus ejaculatorius, and the adjacent ventral side of the tubus of the median lobe with internal sac and its structures (this part is referred to as the parameral side in some recent publications); the opposite side is referred to as the dorsal part. In the species descriptions, microsculpture refers to the surface of the upper forebody (head, pronotum and elytra).

The morphology of antennae and mandibles, body proportions, density of punctures on the forebody, and the shape of the median lobe of the aedeagus and the spermatheca provide the best characteristics for species identification in *Gnathusa*, *Mniusa* and *Ocyusa*.

### Depository/institutional abbreviations

AAFC Agriculture and Agri-Food Canada, Atlantic Cool Climate Crop Research Centre, St. John’s, Newfoundland and Labrador, Canada.

AMNH American Museum of Natural History, New York, New York, USA.

CNC Canadian National Collection of Insects, Arachnids and Nematodes, Agriculture and Agri-Food Canada, Ottawa, Ontario, Canada.

ECW Environment Canada, Whitehorse, Yukon, Canada.

LFC Natural Resources Canada, Canadian Forest Service, Laurentian Forestry Centre, R. Martineau Insectarium, Quebec City, Quebec, Canada.

MUN Memorial University Collection, St. John’s, Newfoundland and Labrador, Canada [on long-term loan to David Langor at NoFC].

NoFC Natural Resources Canada, Canadian Forest Service, Northern Forestry Centre, Edmonton, Alberta, Canada.

NSPM Nova Scotia Provincial Museum, Halifax, Nova Scotia, Canada.

RWC Reginald Webster Private Collection, Charters Settlement, New Brunswick, Canada.

USNM United States National Museum, Washington, D.C., USA.

ZMB Zoological Museum of Humboldt University, Berlin, Germany.

### Key distinguishing *Gnathusa* from *Mniusa* and *Ocyusa*

**Table:** 

1	Mandibles extremely long and narrow, distinctly longer than width of labrum, and sickle-shaped with apices long and very slender, crossing each other in resting position, right mandible bearing a spine and the left a small tooth (Figs 1h, i, 2i, j, 3h, i, 4h, i); frontal suture between eyes absent; ligula more or less deeply bilobed (Figs 1l, 2m, 3l, 4l); anterior margin of mesosternum with short V-shaped basal carina	***Gnathusa* Fenyes**
–	Mandibles moderately long and broad, each as long as the width of labrum or only insignificantly longer (Figs 5h, i, 6h, i, 7h, i, 8h, i, 9h, i), apices gradually narrowed and pointed, right mandible bearing a small tooth and the left a slightly less developed one; frontal suture between eyes present (*Ocyusa*) or absent (*Mniusa*); ligula shallowly split apically (Figs 5l, 6l, 7l, 8l, 9l); anterior margin of mesosternum without V-shaped basal carina	**2**
2	All pronotal setae distributed along midline of pronotum directed posteriad; fronto -clypeal phragma present and visible externally as frontal suture between antennal pits (seen better in diffused light); spermatheca S-shaped with spherical capsule bearing deep and broad invagination and sinuate moderately broad stem (Figs 8e, 9e); tubus of median lobe strongly produced ventrally in lateral view (Figs 8b, 9b); Canadian species are known from riparian habitats	***Ocyusa* Kraatz**
–	Pronotal setae along midline of pronotum directed anteriad in about apical third of midline and posteriad elsewhere; frontal suture absent; spermathecal capsule spherical or tubular and narrowly extended forming L-shaped neck, connected to thin and elongate stem (Figs 5e, 6e, 7e); tubus of median lobe approximately straight in lateral view and slightly produced ventrally (Figs 5b, 6b, 7b); Canadian species are known from forests	***Mniusa* Mulsant & Rey**

## Taxonomic review

### Gnathusa

Fenyes

http://species-id.net/wiki/Gnathusa

Gnathusa Fenyes 1909: 197. Type species: *Gnathusa eva* Fenyes.

#### Diagnosis.

**Figure 1.**
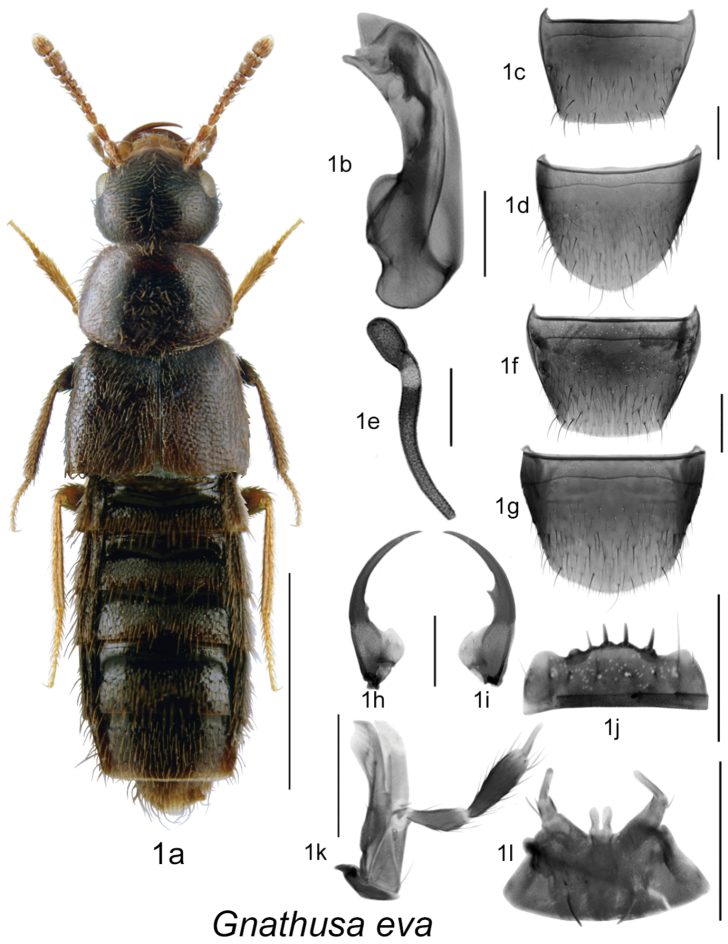
*Gnathusa eva* Fenyes: **1a** habitus **1b** median lobe of aedeagus in lateral view **1c** male tergite VIII **1d** male sternite VIII **1e** spermatheca in lateral view **1f** female tergite VIII **1g** female sternite VIII **1h** left mandible **1i** right mandible **1j** labrum **1k** maxilla **1l** menthum, labial palpi and ligula. Habitus scale bar = 1.0 mm; other scale bars = 0.2 mm.

**Figure 2.**
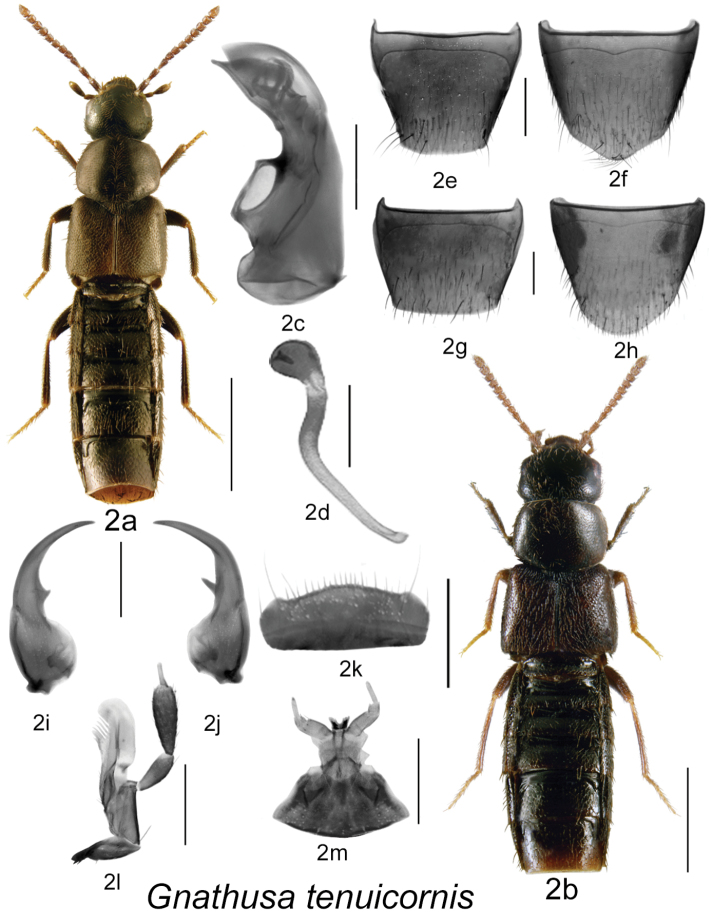
*Gnathusa tenuicornis* Fenyes: **2a** habitus, based on female from New Brunswick **2b** habitus based on male from Alberta **2c** median lobe of aedeagus in lateral view **2d** spermatheca in lateral view **2e** male tergite VIII **2f** male sternite VIII **2g** female tergite VIII **2h** female sternite VIII **2i** left mandible **2j** right mandible **2k** labrum **2l** maxilla **2m** menthum, labial palpi and ligula. Habitus scale bar = 1.0 mm; other scale bars = 0.2 mm.

**Figure 3.**
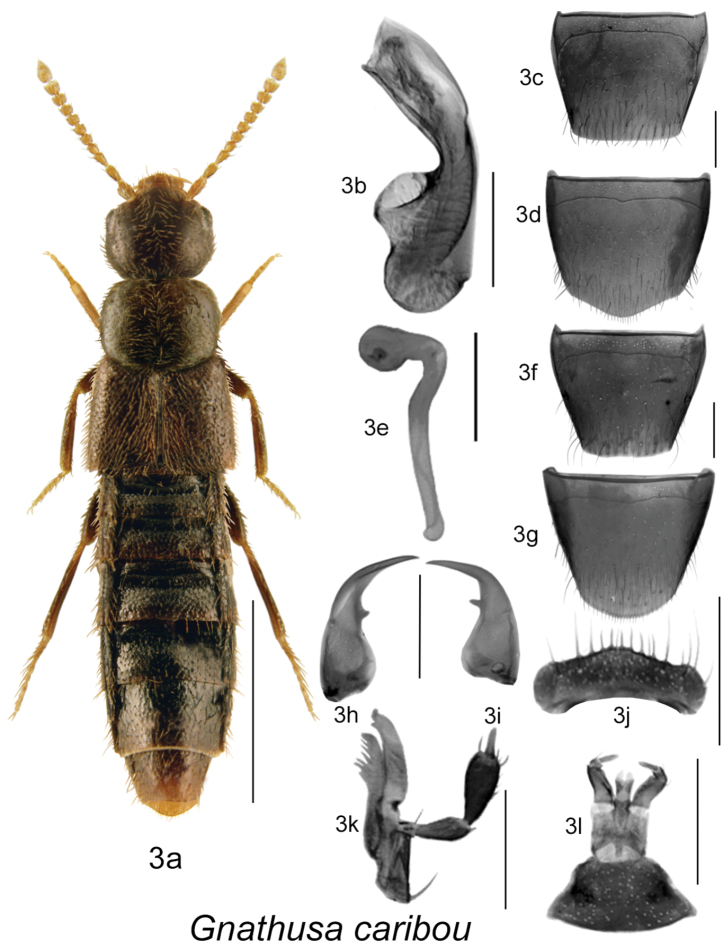
*Gnathusa caribou* Lohse: **3a** habitus **3b** median lobe of aedeagus in lateral view **3c** male tergite VIII **3d** male sternite VIII **3e** spermatheca in lateral view **3f** female tergite VIII **3g** female sternite VIII **3h** left mandible **3i** right mandible **3j** labrum **3k** maxilla **3l** menthum, labial palpi and ligula. Habitus scale bar = 1.0 mm; other scale bars = 0.2 mm.

**Figure 4.**
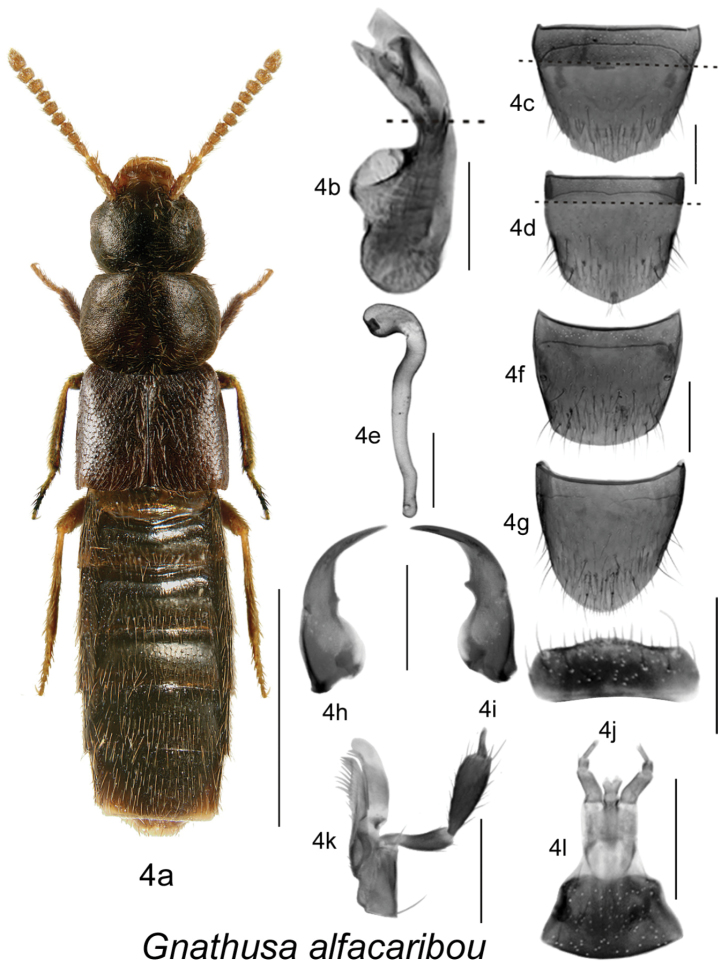
*Gnathusa alfacaribou* Klimaszewski and Langor: **4a** habitus **4b** median lobe of aedeagus in lateral view, partially reconstructed below broken line in Fig. **4b**, and above broken line in Fig. **4c, 4d**; based on the holotype **4c** male tergite VIII partially reconstructed above broken line, based on the holotype **4d** male sternite VIII partially reconstructed above broken line, based on the holotype **4e** spermatheca in lateral view **4f** female tergite VIII **4g** female sternite VIII **4h** left mandible **4i** right mandible **4j** labrum **4k** maxilla **4l** menthum, labial palpi and ligula. Habitus scale bar = 1.0 mm; other scale bars = 0.2 mm.

Body dark brown to black, compact and robust, subparallel (Figs 1a, 2a, b, 3a, 4a), length 3.5–4.3 mm; integument with distinct meshed microsculpture; head large with mandibles extremely long and sickle-shaped, apices very slender, each crossing the other in resting position, each mandible bearing long spine or tooth (Figs 1h, i, 2i, j, 3h, i, 4h, i); infraorbital carina strong and complete; ligula more or less deeply bilobed (Figs 1l, 2m, 3l, 4l); last article of maxillary palpus needle-shaped (Figs 1k, 2l, 3k, 4k); frontal suture of head absent; anterior margin of mesosternum with short V-shaped basal carina, remaining mesosternum uncarinated; mesosternal process long, triangular basally and then narrowly produced and extending to about ¾ length of mesosternal cavities, metasternal process triangular in shape and short, isthmus short; median lobe of aedeagus with large crista apicalis, internal sac structures variable in shape (Figs 1b, 2c, 3b, 4b), paramere with narrowly elongate apical lobe bearing four macrosetae; spermatheca pipe-shaped with spherical or tubular capsule and elongate narrow stem (Figs 1e, 2d, 3e, 4e).

#### Key to Canadian species of *Gnathusa*

New provincial and territorial records are indicated in boldface font.

**Table:** 

1	Labrum with coarse spines (Fig. 1j); antennal articles 7–10 strongly transverse (Fig. 1a); body length 3.5–4.0 mm; integument moderately glossy; genitalic structures as illustrated (Fig. 1b, e)	***Gnathusa eva* Fenyes** [AB, BC, CA, YT]
–	Labrum with fine setae (Figs 2k, 3j, 4j); antennal articles 7-10 subquadrate or slightly transverse (Figs 2a, b, 3a, 4a); body length 2.5–3.7 mm; integument usually more glossy; genital structures differently shaped (Figs 2c, d, 3b, e, 4b, e)	**2**
2(1)	Antennal articles 5–10 subquadrate (Fig. 2a, b); genitalia as illustrated (Figs 2c, d); body length 2.5–3.7 mm	***Gnathusa tenuicornis* Fenyes** [**AB**, AK, BC, YT, **OR**, **NB**]
–	Antennal articles 5–10 slightly transverse (Figs 3a, 4a); genitalia differently shaped (Figs 3b, e, 4b, e); body length 2.8–3.6 mm	**3**
3(2)	Head slightly narrower and smaller than pronotum in dorsal view (Fig. 4a); antennae brown or yellowish-brown; basal part of abdominal tergite III deeply impressed and moderately coarsely-densely punctate; genitalia as illustrated (Fig. 4b, e)	***Gnathusa alfacaribou* Klimaszewski & Langor** [NF, LB]
–	Head about as wide and large as pronotum in dorsal view (Fig. 3a); antennae light yellow; basal part of abdominal tergite III deeply impressed and coarsely-densely punctate; genitalia as illustrated (Figs 3b, e; Figs 45–47, in Lohse et al. 1990)	***Gnathusa caribou* Lohse** [YT, NWT, AK]

### 1. Gnathusa eva

Fenyes

http://species-id.net/wiki/Gnathusa_eva

Figure 1a–l , Map 1

Gnathusa eva Fenyes 1909: 198, 1920: 352, Moore and Legner 1975: 458, Majka and Klimaszewski 2008: 88.

#### Diagnosis.

Body length 3.5–4.0 mm, sides subparallel; body colour light brown to dark brown, antennae and tarsi rust-brown, head and abdomen often dark brown; integumental microsculpture dense and surface moderately glossy; head round and almost as wide as pronotum with labrum bearing long spines; pronotum transverse, angular, wider than maximum width of elytra; elytra at suture subequal in length to pronotum; abdomen subparallel; antennal articles 6-10 moderately-to-strongly transverse, last article short and broadly oval (Fig. 1a). MALE: tergite VIII widely truncate apically (Fig. 1c); sternite VIII slightly pointed at apex (Fig. 1d); median lobe of aedeagus with tubus arcuate and apex pointed and produced ventrally in lateral view (Fig. 1b). FEMALE: tergite VIII truncate apically (Fig. 1f); sternite VIII rounded apically (Fig. 1g); spermatheca pipe-shaped, with short sac-shaped capsule and long, thin and sinuate stem, spermathecal neck weakly sclerotized and neck to capsule angle variable (Fig. 1e).

#### Distribution.

**Maps 1–2.**
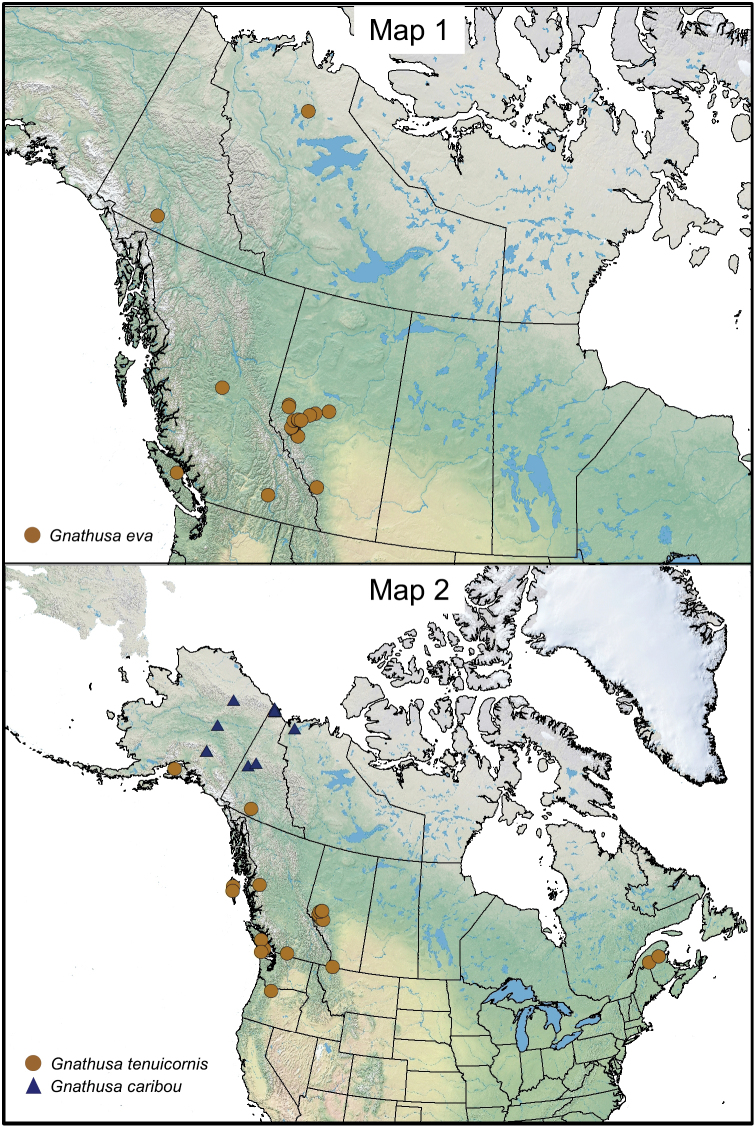


The Canadian distribution of this native Nearctic species includes Alberta [new provincial record], British Columbia (Majka and Klimaszewski 2008), and Yukon Territory (Map 1). It is also known from California (Fenyes 1909).

#### Bionomics.

Adults were captured in clear-cut Sitka spruce forest on Vancouver Island and in moss and gravel at the edge of small pools at other localities in the interior of British Columbia (Klimaszewski and Winchester 2002). Additional specimens were found in British Columbia in a 1-year-old harvested Douglas-fir stand. In west-central Alberta, adults were collected in pitfall traps deployed in Upper Cordilleran coniferous forests, including subxeric lodgepole pine forests, mesic white spruce and lodgepole pine stands and spruce-dominated subhygric and hygric forests, but not in deciduous-dominated forest or in grassy or shrubby meadows. In Alberta, adults also emerged from lodgepole pine trees infested by bark beetles. In the Yukon Territory, adults were found in a squirrel midden in spring, probably overwintering, and in a coniferous woodchip pile.

#### Locality data.

CANADA: **Alberta:** Lusk Creek, Kananaskis F.E.S., 14.VII.1971, J.M. & B.A. Campbell (CNC) 1 male, 4 females; vicinity of Swan Hills, 54°42'N, 115°23'W, *Picea*/*Pinus* forest, 15.VI.1990, D.W. Langor (NoFC) 2 males; Grande Prairie, 64 km S, 54.5597°N, 118.6633°W, emergence trap on MPB infested lodgepole pine, 14 July 2011, col. Bleiker (NoFC) 1 female; same data except 15 July 2011, 1 female, 1 sex undetermined; Grande Prairie, 75 km S, 54.4706°N, 118.6560°W, 13 Aug. 2011, emergence trap on MPB infested lodgepole pine, col. Bleiker (NoFC) 1 sex undetermined; Fox Creek, 24 km E, 54.4575°N, 116.4377°N, emergence trap on MPB infested lodgepole pine, 9 Aug. 2011, col. Bleiker (NoFC) 1 female; Fox Creek, 7 km SW, 54.3241°N, 116.8335°W, emergence trap on MPB infested lodgepole pine, 18 July 2011, col. Bleiker (NoFC) 1 sex undetermined; 20 km NW Hinton, 4 km NW of Jarvis Lake, 53.484°N, 117.854°W, Ecosite Surrogacy Study, Ecoregion: UF, Ecosite I1, Stand I103, pitfall trap # 4, 19.VI–3.VII.2004, J. Hammond et al. coll. (NoFC) 1 sex undetermined; 20 km S Hinton, 31.V.1990, pitfall trap, D. Langor (NoFC) 1 sex undetermined; 21.3 km NW Hinton, W.A. Switzer Prov. Pk., 53.529°N, 117.824°W, Ecosite Surrogacy Study, Ecoregion: UF, Ecosite E1, Stand E129, pitfall trap # 5, 3–17.VII.2004, J. Hammond et al. coll. (NoFC) 1 female; 23 km NW Hinton, 1.7 km W of Gregg Lake, 53.545°N, 117.821°W, Ecosite Surrogacy Study, Ecoregion: UF, Ecosite H1, Stand H101, pitfall trap # 1, 2–16.VII.2004, J. Hammond et al. coll. (NoFC) 1 female; 25 km NW Hinton, west side Hay River Rd., 53.502°N, 117.909°W, Ecosite Surrogacy Study, Ecoregion: UF, Ecosite C1, Stand C101, pitfall trap # 4, 2–17.VII.2004, J. Hammond et al. coll. (NoFC) 1 male; same data except: pitfall trap # 6, 17–31.VII.2004, J. Hammond et al. coll. (NoFC) 1 sex undetermined; 26 km SE Hinton, 7 km S of Gregg River Rd., 53.220°N, 117.343°W, Ecosite Surrogacy Study, Ecoregion: UF, Ecosite H1, Stand H104, pitfall trap # 1, 16.V–1.VI.2004, J. Hammond et al. coll. (NoFC) 2 sex undetermined; 32 km NW Hinton, 1 km W of Rock Lake Rd., 53.561°N, 117.998°W, Ecosite Surrogacy Study, Ecoregion: UF, Ecosite F1, Stand F104, pitfall trap # 1, 2–16.VI.2004, J. Hammond et al. coll. (NoFC) 1 male, 5 sex undetermined; 33 km NW Hinton, 3.75 km N of Highway 40, 53.594°N, 117.964°W, Ecosite Surrogacy Study, Ecoregion: UF, Ecosite E1, Stand E108, pitfall trap # 4, 4–18.VI.2004, J. Hammond et al. coll. (NoFC) 1 female, 1 sex undetermined; 34 km NW Hinton, 0.5 km W of Highway 40, Ecosite Surrogacy Study, Ecoregion: UF, Ecosite F1, Stand F102, pitfall trap # 4, 4–18.VI.2004, J. Hammond et al. coll. (NoFC) 3 sex undetermined; 35 km NW Hinton, 3.75 km N of Highway 40, 53.596°N, 118.002°W, Ecoregion: UF, Ecosite D1, Stand D109, pitfall trap # 4, 2–16.VII.2004, J. Hammond et al. coll. (NoFC) 1 female; 36 km NW Hinton, 3.75 W of Rock Lake Rd., 53.564°N, 118.046°W, Ecosite Surrogacy Study, Ecoregion: UF, Ecosite I1, Stand I107, pitfall trap # 4, 14.V–4.VI.2004, J. Hammond et al. coll. (NoFC) 1 female; 59 km NW Hinton, 3.5 km N of Polecat Rd., 53.902°N, 117.911°W, Ecosite Surrogacy Study, Ecoregion: UF, Ecosite H1, Stand H105, pitfall trap # 2, 3–17.VI.2004, J. Hammond et al. coll. (NoFC) 1 sex undetermined; 62 km N Hinton, 5 km W of J. Wright Rd., 53.969°N, 117.668°W, Ecosite Surrogacy Study, Ecoregion: UF, Ecosite F1, Stand F105, pitfall trap # 2, 12.V–2.VI.2004, J. Hammond et al. coll. (NoFC) 1 female, 1 sex undetermined; 63 km N Hinton, 3.75 km S of J. Wright Rd., 53.974°N, 117.449°W, Ecosite Surrogacy Study, Ecoregion: UF, Ecosite D1, Stand D102, pitfall trap # 2, 30.VI–14.VII.2004, J. Hammond et al. coll. (NoFC) 1 male, 1 female; 65 km N Hinton, 5 km W of J. Wright Rd., 53.995°N, 117.656°W, Ecosite Surrogacy Study, Ecoregion: UF, Ecosite E1, Stand E118, pitfall trap # 5, 30.VI–14.VII.2004, J. Hammond et al. coll. (NoFC) 1 sex undetermined; 67 km N Hinton, north side of J. Wright Rd., 53.998°N, 117.435°W, Ecosite Surrogacy Study, Ecoregion: UF, Ecosite D1, Stand D104, pitfall trap # 2, 2–16.VI.2004, J. Hammond et al. coll. (NoFC) 1 sex undetermined. **British Columbia:** Fort St. James, PG13B-trap 4, 10.V. 1995 (CNC) 1 female; same data except: GP 11 km-2, 1 year post harvest (CNC) 1 male; GP 115, 30.V.1996, 1 year post harvest, R. Felix (CNC) 1 male; 20.VI.1920, GP 11 km, 2 years post harvest, D. Rodriguez (CNC) 1 female; 4.VIII.1996, Tachie-Pinchi, M. Cloet, trap 5 (CNC) 1 female; GP 11 km-2, 10.V.1995, 1 year post harvest (CNC) 3 females; 21 km SW Campbell River, 49°51'55"N, 125°27'51"W, 22.V-6.VI.1996, Balsam Cr., LT 1-T, 1-E (LFC) 2 males; Monashee Mountain near Cherryville, 12.VIII.1982, R. Baranowski (LFC, MZLU) 1 female, 1 sex undetermined [published record Majka and Klimaszewski 2008]. **Yukon Territory:** Whitehorse, Paddy’s Pond, 15.V.2010, 60.7067°N, 135.0917°W, 649 m, soil sifting, squirrel midden, B. Godin (ECW) 2 males, 3 females; Whitehorse, Granger subdivision, coniferous woodchip pile, 2.IX.2007, 60.7097°N, 135.0996°W, 661 m, pitfall trap, B. Godin (ECW) 1 male; same data as before except: 3.V.2008 (ECW) 2 males.

UNITED STATES OF AMERICA: **California:** Mono Co., 6 mi SW Toms Place, 9000’, 8.VIII.1969, A. Smetana (CNC) 3 males, 1 female [not shown in Map 1].

### 2. Gnathusa tenuicornis

Fenyes

http://species-id.net/wiki/Gnathusa_tenuicornis

Figure 2a–m , Map 2

Gnathusa tenuicornis Fenyes 1921: 26, Moore and Legner 1975: 458, Klimaszewski and Winchester 2002: 58.

#### Diagnosis.

Body length 2.5–3.7 mm, sides subparallel; body colour light brown to almost black, with antennae, tarsi and often elytra and apical part of abdomen rust-brown; integumental microsculpture dense and surface strongly glossy; head round (Fig. 2a) to somewhat quadrate (Fig. 2b) and almost as wide as pronotum, labrum with short fine setae but lacking coarse spines; pronotum transverse, subequal to slightly narrower than maximum width of elytra, corners somewhat angular; elytra at suture subequal in length to pronotum; abdomen subparallel; antennal articles 6–10 subquadrate, last article short and broadly oval (Fig. 2a, b). MALE: tergite VIII widely truncate apically (Fig. 2e); sternite VIII slightly pointed at apex (Fig. 2f); median lobe of aedeagus with tubus almost straight and apex pointed ventrally in lateral view (Fig. 2c). FEMALE: tergite VIII truncate apically (Fig. 2g); sternite VIII rounded apically (Fig. 2h); spermatheca pipe-shaped, with spherical capsule and long, thin, and slightly sinuate stem, neck weakly sclerotized, and neck to capsule angle variable (Fig. 2d).

#### Distribution.

This native Nearctic species was described from specimens captured in Glacier, British Columbia, later recorder from Yokon, and is herein recorded for the first time from Alberta and New Brunswick (four female specimens tentatively identified as this species) (Map 2). In the United States, this species was previously known from California (Fenyes 1921, Moore and Legner 1975, Klimaszewski and Winchester 2002), and is herein recorded for the first time from Oregon.

#### Bionomics.

Adults were captured in a clear-cut Sitka spruce forest on Vancouver Island and in moss and gravel at the edge of small pools in British Columbia (Klimaszewski and Winchester 2002). Other adults were found in a subalpine meadow at 3000 feet, in cold moss and gravel along the edges of streams. The Yukon specimens were taken from mixed aspen and spruce forest by sifting litter. In west-central Alberta, adults were collected in pitfall traps deployed in Upper Cordilleran coniferous forests, including subxeric lodgepole pine forests, mesic white spruce and lodgepole pine stands and and spruce-dominated subhygric and hygric forests, but not in deciduous-dominated forest or in grassy or shrubby meadows. The New Brunswick specimens were taken from moss and leaves under alders near a brook in an eastern white-cedar swamp and from under cobblestones and gravel in sand on a partially shaded cobblestone bar near the outflow of a brook into a river. Adults were captured from May through August.

#### Locality data.

CANADA: **Alberta:** Waterton Lakes National Park, Cameron Lake, 5450’, 4.VIII.1976, J.M. Campbell (CNC) 1 male; 20 km S Hinton, 20.VII.1989, pitfall trap, D. Langor coll., site C, trap 6, conifer study (NoFC) 1 male; 20 km S Hinton, 26.VI.1989, D. Langor coll., site C, trap 6, conifer study (NoFC) 1 male; 26 km SE Hinton, 7 km S of Gregg River Rd., 53.220°N, 117.343°W, Ecosite Surrogacy Study, Ecoregion: UF, Ecosite H1, Stand H104, pitfall trap # 4, 15–29.VI. J. Hammond et al. (NoFC) 2 males, 1 female; 31 km SE Hinton, 3 km of Highway 40, 53.593°N, 117.925°W, Ecosite Surrogacy Study, Ecoregion: UF, Ecosite D1, Stand D108, pitfall trap # 6, 11.V-3.VI. J. Hammond et al. (NoFC) 1 male; 32 km NW Hinton, 3 km W of Highway 40, 53.586°N, 117.954°W, Ecosite Surrogacy Study, Ecoregion: UF, Ecosite E1, Stand E103, pitfall trap # 5, 11.V-4.VI. J. Hammond et al. (NoFC) 1 male; 32 km NW Hinton, 1 km W of Hay River Rd., 53.760°N, 117.652°W, Ecosite Surrogacy Study, Ecoregion: UF, Ecosite I102, Stand I102, pitfall trap # 2, 13.V–3.VI. 2004, J. Hammond et al. (NoFC) 1 male, 1 female; 43 km SE Hinton, 1.5 km N Coalspur, 53.194°N, 117.046°W, Ecosite Surrogacy Study, Ecoregion: UF, Ecosite B1, Stand B103, pitfall trap # 6, 1–15.VI.2004, J. Hammond et al. (NoFC) 1 male; 55 km N Hinton, north side of Polecat Rd., 53.855°N, 117.926°W, Ecosite Surrogacy Study, Ecoregion: UF, Ecosite H1, Stand H103, pitfall trap # 3, 3–17.VI.2004, J. Hammond et al. (NoFC) 1 male, 1 female; 65 km N Hinton, 5 km W of Wright Rd., 53.995°N, 117.656°W, Ecosite Surrogacy Study, Ecoregion: UF, Ecosite E1, Stand E118, pitfall trap # 5, 2–16.VI.2004, J. Hammond et al. (NoFC) 2 females; 69.5 km N Hinton, 0.7 km NW of JV Haul Rd., 54.017°N, 117.618°W, Ecosite Surrogacy Study, Ecoregion: UF, Ecosite D1, Stand D103, pitfall trap # 5, 1–16.VI.2004, J. Hammond et al. (NoFC) 1 female. **British Columbia:** Glacier, Fenyes collection (CAS) 1 female [holotype]; Nitinat, Heather Mtn., subalpine meadow at 3000’, 14.VII.1979, I.M. Smith, moss on seepage slope (CNC) 1 female; Forbidden Plateau, Murray Meadows, 3400’, 21.VII.1975, J.M. and B.A. Campbell (CNC) 1 female; Queen Charlotte Islands, 10.5 km NW Rennell, Sound Rd., Ghost Main Rd., 900’, J.M. Campbell, cold moss along stream (CNC) 1 male; Queen Charlotte Islands, Moresby Is., Mt. Moresby, 25.VII.1983, 2100’, J.M. Campbell, ex gravel at edge of stream (CNC) 1 male; 20 mi E Hope, Manning Pk., 21.VI.1968, Campbell and Smetana (CNC) 1 female; Copper River Valley, A37574/P4–1-1, 6.VI–5.VII.1996, pitfall trap, J. Lemieux (LFC) 1 female; same data except: 5.VII–12.VIII.1996, (LFC) 1 male; Upper Carmanah Valley, UTM: 10U CK 803005, 16.VII-30.VII.1991, CC MT3, N. Winchester (LFC) 2 females [additional records from the same locality Klimaszewski and Winchester 2002]. **Yukon Territory:** Whitehorse, Paddy’s Pond, 6.V.2007, 60.7067°N, 135.0917°W, 649 m, litter sifting, mixed aspen and spruce forest, B. Godin (ECW) 1 male [record from Klimaszewski et al. 2012]. **New Brunswick:** Restigouche Co., MacFarlane Brook Protected (Natural) Area, 47.6018°N, 67.6263°W, 25.V.2007, R.P. Webster // old growth eastern white cedar swamp, in moss & leaves under alders near stream (RWC) 2 females; Jacquet River Gorge PNA, 47.8257°N, 66.0779°W, 24.V.2010, R.P. Webster // partially shaded cobblestone bar near outflow of brook at Jacquet River, under cobblestones & gravel on sand (RWC) 1 female; Mount Atkinson, 441 m elev., 41.8192°N, 68.2618°W, 7.VII.2011, R.P. Webster // Boreal forest, small shaded spring-fed brook with mossy margin, sifting moss (LFC) 1 female.

UNITED STATES OF AMERICA: **Alaska:** Kenai Peninsula, 2 mi NE Soldotna, 10.VI.1978, Smetana and Becker (CNC) 1 female. **Oregon:** Mt. Hood, Timberline Lodge Road, 4500–5000’, 28.VI.1974, A. and D. Smetana (CNC) 1 male.

#### Comments.

We have tentatively included the females from New Brunswick as belonging to this species. The difference in body colour, the slightly different shape of pronotum and the temples of the head in the New Brunswick and western specimens we attribute to infraspecific variations because the shape of spermatheca and the tergites and sternite VIII are similar in females of both populations. The study of males from New Brunswick is critical to confirm our identification. The specimens from the north usually are darker than the specimens from more southern localities in many species of aleocharines.

### 3. Gnathusa caribou

Lohse

http://species-id.net/wiki/Gnathusa_caribou

Figure 3a–l , Map 2

Gnathusa caribou Lohse, in Lohse et al. 1990: 146; Klimaszewski et al. 2011: 55.

#### Diagnosis.

Body length 2.8–3.6 mm, sides narrowly subparallel; body colour dark brown to almost black, with antennae bright yellow and tarsi rust-brown to yellowish; integumental microsculpture dense and surface strongly glossy; head round, about the same size as the pronotum, labrum lacking stout spines but with fine setae of unequal length; pronotum small, transverse, angular, slightly narrower than elytra; elytra at suture subequal in length to pronotum; abdomen subparallel; antennal articles 6–10 subquadrate to slightly transverse, last article short and broadly oval (Fig. 3a). MALE: tergite VIII widely truncate apically (Fig. 3c); sternite VIII slightly pointed at apex (Fig. 3d); median lobe of aedeagus with tubus almost straight and apex pointed ventrally in lateral view (Fig. 3b). FEMALE: tergite VIII truncate apically (Fig. 1f); sternite VIII rounded apically (Fig. 3g); spermatheca pipe-shaped, with spherical capsule and long and straight stem, neck well sclerotized (Fig. 3e).

#### Distribution.

This native Nearctic species is known in Canada from the Northwest Territories and Yukon Territory (Map 2), and from Alaska (Lohse et al. 1990).

#### Bionomics.

Adults were captured from June to July in tundra by sifting organic litter under *Salix*, moss, and a pile of leaves stored by a rodent.

#### Locality data.

CANADA: **Northwest Territories:** Lac Maunoir, North shore, 19–27.VII.1969, G.E. Shewell (CNC) 1 male; Reindeer Sta., Caribou Hills, 2.VII.1972, A. Smetana (CNC) 1 male, 2 females, 1 sex undetermined [paratypes]; same label data except: 30.VI.1972 (CNC) 1 female [paratype]. **Yukon Territory:** British Mts., Firth River, 250 m, 69°13'N, 140°04'W, 25.VI.1984, 84–31, tundra, sifting litter under *Salix* (CNC) 1 male [holotype]; same label data (CNC) 1 female, 3 sex undetermined [paratypes]; British Mts., Windy Ridge, 550 m, 69°27'N, 140°25'W, 2.VII.191984, 84–46, sifting moss, J.M. Campbell (CNC) 4 sex undetermined [paratypes]; British Mts., Fish Creek, 200 m, 69°27'N, 140°23'W, 5.VII.1984, 84–58, sifting moss and arctic willow on tundra, J.M. Campbell (CNC) 1 female, 4 sex undetermined [paratypes]; British Mts., Sunday Mts., 680 m, 69°14'N, 140°05'W, 24.VI.1984, sifting pile of leaves stored by rodent, J.M. Campbell (CNC) 1 male, 2 females, 4 sex undetermined [paratypes]; Dawson City, 11.VII.1968, Campbell and Smetana (CNC) 1 male, 1 female; Dempster Hwy., mi 53, North Fork Pass, 24.VII.1978, 4200’, A. Smetana and J.M. Campbell (CNC) 1 female, 1 sex undetermined [paratypes].

UNITED STATES OF AMERICA: **Alaska:** Prudhoe Bay Rd., 9 mi N Atigun Pass, 68°14'N, 149°25'W, 6.VII.1978, 3100’, J.M. Campbell and A. Smetana (CNC) 1 female, 3 sex undetermined [paratypes]; mi 104.5 Denali Hwy., Brushkana Cr., 15.VII.1978, A. Smetana and J.M. Campbell (CNC) 1 sex undetermined [paratype]; mi 110 Denali Hwy., Seattle Cr., 15.VII.1978, J.M. Campbell and A. Smetana (CNC) 1 male [paratype]; mi 24, Wales Hwy., Hess Cr., 600’, 65°40'N, 149°10'W, 10.VII.1978, J.M. Campbell and A. Smetana (CNC) 1 male [non-paratype].

### 4. Gnathusa alfacaribou

Klimaszewski & Langor

http://species-id.net/wiki/Gnathusa_alfacaribou

Figure 4a–l , Map 3

Gnathusa alfacaribou Klimaszewski et al. 2011: 55–56.

#### Diagnosis.

Body length 3.0–3.4 mm, sides subparallel; body colour dark brown, with tarsi lighter and antennae brown and often with reddish tinge; head round and almost as wide as pronotum or at most as wide as pronotum, equal in size to pronotum; pronotum transverse, angular, about as wide as maximum width of elytra; abdomen subparallel, at base as wide as elytra, widest in apical half; antennal articles 5–10 quadrate to slightly transverse (Fig. 4a). MALE: tergite VIII pointed apically (Fig. 4c); sternite VIII slightly pointed at apex (Fig. 4d); median lobe of aedeagus with tubus strongly produced ventrally in lateral view, apex pointed (Fig. 4b). FEMALE: tergite VIII truncate apically (Fig. 4f); sternite VIII rounded apically (Fig. 4g); spermatheca pipe-shaped, with small spherical capsule and long, thin and almost straight stem (Fig. 4e).

#### Distribution.

**Maps 3–4.**
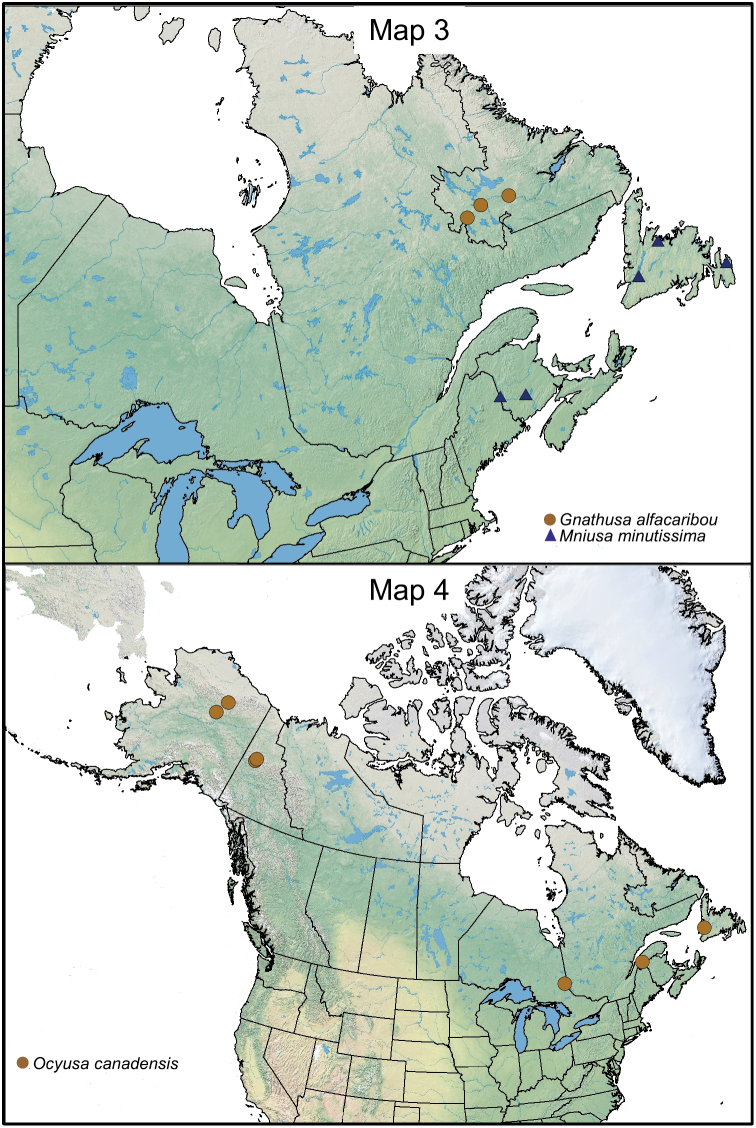


This native Nearctic species is known only from Labrador (Map 3).

#### Bionomics.

This epigaeic species was collected from June to October using pitfall and flight intercept traps in black spruce-lichen, spruce-moss and old fir forests.

#### Locality data.

CANADA: **Newfoundland:** Labrador, Middle Brook, Lake Melville, Plot: MID 4, 17.VI.2005 (LFC) 1 male [holotype]; Labrador, Middle Brook, Lake Melville, Plot: MID 4, 17.VI.2005 (MUN) 1 male [paratype]; same data except: Plot: MID 3, 4.VII.2005 (MUN) 1 female [paratype], Plot: MID 4 (MUN) 1 female [paratype], Plot: MID 5, 18.VII.2005 (MUN) 1 female [paratype]; Labrador, Ossak Camp, Station 1, lichen-black spruce forest, 8.X.2004 (MUN) 1 female [paratype]; SW Labrador, 72 km E Labrador City, Rt. 500, km 93, 53°08.6 N, 66°05.9 W, 12–27.VIII.2001, S. and J. Peck, FIT, 600 m, spruce-moss forest 2001-34 (LFC) 1 female [paratype].

### Mniusa

Mulsant & Rey

http://species-id.net/wiki/Mniusa

Mniusa Mulsant & Rey, 1875. Type species: *Homalota incrassata* Mulsant & Rey.

#### Diagnosis.

**Figure 5.**
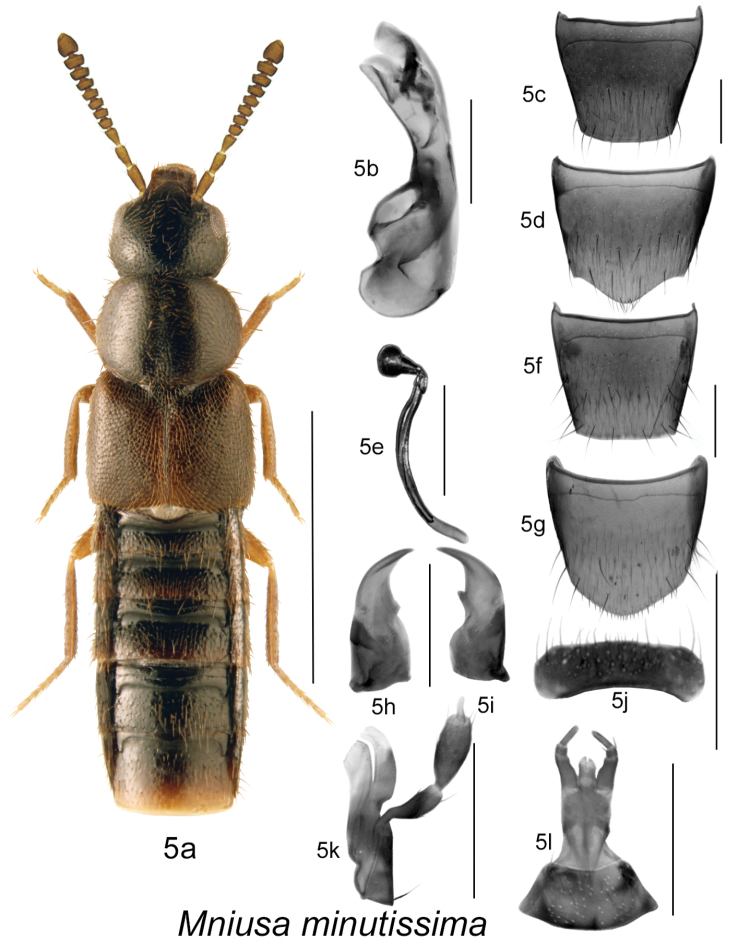
*Mniusa minutissima* (Klimaszewski & Langor): **5a** habitus **5b** median lobe of aedeagus in lateral view **5c** male tergite VIII **5d** male sternite VIII **5e** spermatheca in lateral view **5f** female tergite VIII **5g** female sternite VIII **5h** left mandible **5i** right mandible **5j** labrum **5k** maxilla **5l** menthum, labial palpi and ligula. Habitus scale bar = 1.0 mm; other scale bars = 0.2 mm.

**Figure 6.**
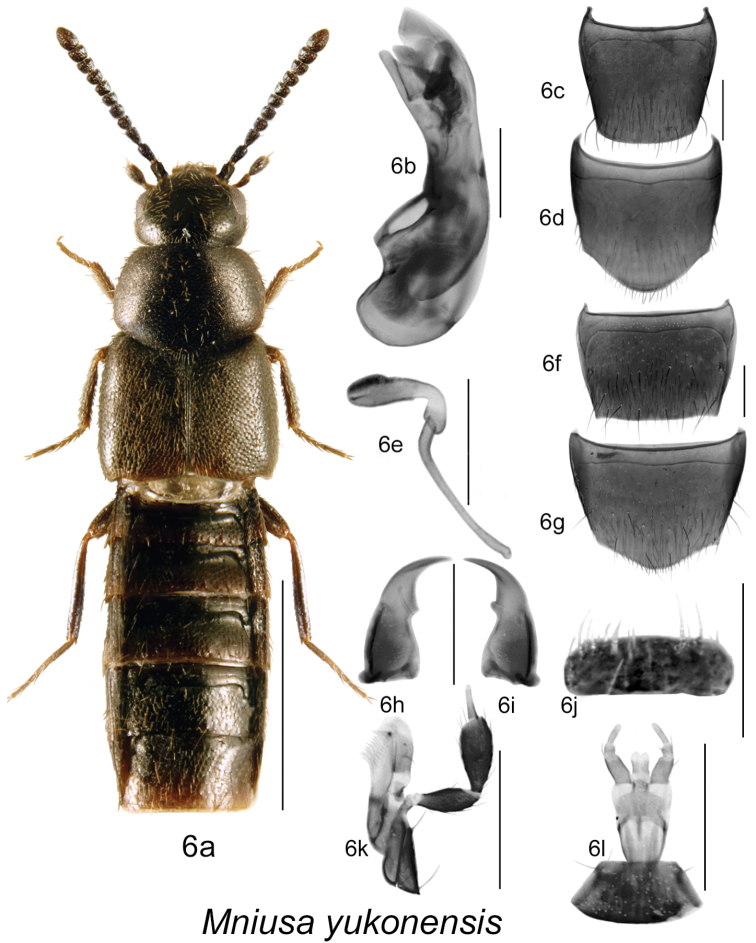
*Mniusa yukonensis* (Klimaszewski and Godin): **6a** habitus **6b** median lobe of aedeagus in lateral view **6c** male tergite VIII **6d** male sternite VIII **6e** spermatheca in lateral view **6f** female tergite VIII **6g** female sternite VIII **6h** left mandible **6i** right mandible **6j** labrum **6k** maxilla **6l** mentum, labial palpi and ligula. Habitus scale bar = 1.0 mm; other scale bars = 0.2 mm.

**Figure 7.**
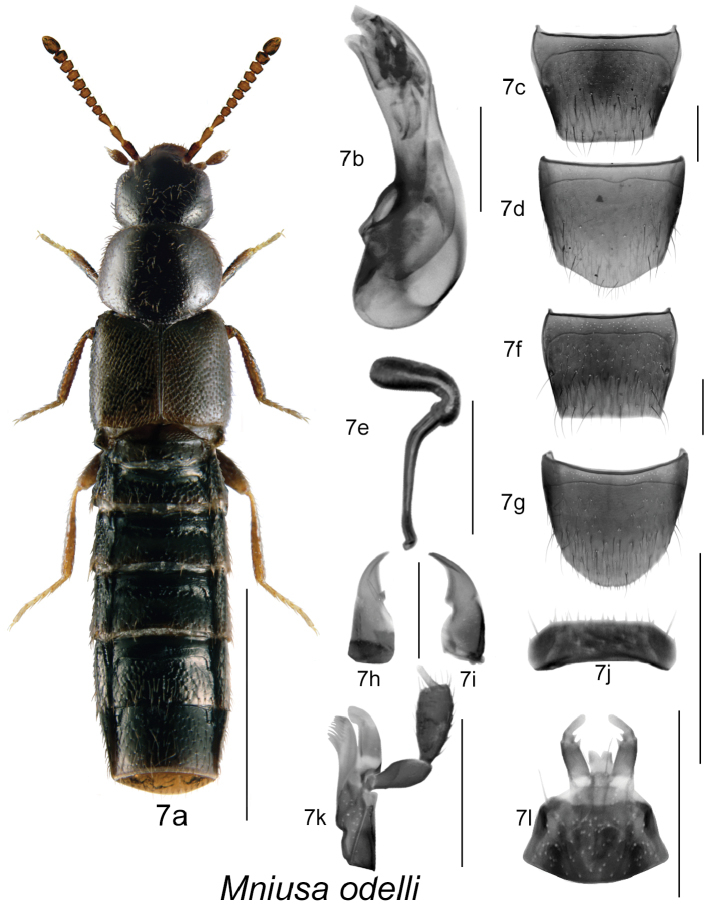
*Mniusa odelli* Klimaszewski and Webster: **7a** habitus **7b** median lobe of aedeagus in lateral view **7c** male tergite VIII **7d** male sternite VIII **7e** spermatheca in lateral view **7f** female tergite VIII **7g** female sternite VIII **7h** left mandible **7i** right mandible **7j** labrum **7k** maxilla **7l** menthum, labial palpi and ligula. Habitus scale bar = 1.0 mm; other scale bars = 0.2 mm.

Body dark brown to black, compact, sides subparallel or body narrowly oval in outline (Figs 5a, 6a, 7a), length 2.0–3.2 mm; integument with distinct meshed microsculpture and moderately dense punctation and pubescence; head large with mandibles broad and long, left mandible with a small tooth (Figs 5h, 6h, 7h), and right one with a slightly larger tooth at the base of arcuate cutting edge of mandible, apices strongly narrowly elongate [more than in *Ocyusa* and less than in *Gnathusa*] (Figs 5h, i, 6h, i, 7h, i); infraorbital carina strong and complete; ligula shallowly split apically (Figs 5l, 6l, 7l); labial palpus with three articles, second article minute, last one needle-shaped (Figs 5l, 6l, 7l), and lacinia and galea as illustrated (Figs 5k, 6k, 7k); labrum narrow and transverse, apical edge entire (Figs 5j, 6j, 7j); frontal suture of head absent; pronotal pubescence along midline directed anteriad or obliquely anteriad in about apical third and posteriad or obliquely posteriad medio-basally; anterior margin of mesosternum without longitudinal carina; mesosternal process triangular basally and then produced and extending from 1/3 to almost 2/3 length of mesosternal cavities, metasternal process triangular in shape and short; isthmus short to long; median lobe of aedeagus strongly produced ventrally, internal sac structures as illustrated (Figs 5b, 6b, 7b); spermatheca with spherical or tubular capsule narrowed posteriorly into L-shaped neck, invagination narrow; stem narrowly elongate (Figs 5e, 6e, 7e).

#### Key to Canadian species of *Mniusa*

New provincial and territorial records are indicated in boldface font.

**Table:** 

1	Antennal articles 7–10 strongly transverse and at least twice as wide as long, terminal article short and conical, no more than twice length of penultimate one (Fig. 5a); genitalia as illustrated (Figs 5b, e)	***Mniusa minutissima* (Klimaszewski & Langor)** [NF, **NB**]
–	Antennal articles 7–10 subquadrate or moderately transverse, terminal article at least twice as long as penultimate one (Figs 6a, 7a); genitalia differently shaped	**2**
2(1)	Body narrowly subparallel, with pronotum basally, elytra, and 2/3 of abdomen subequal in width, head slightly narrower than pronotum, pronotum quadrate or slightly transverse (Fig. 7a); forebody strongly glossy, sparsely punctate and pubescent; elytra at suture about as long as pronotum (Fig. 7a); median lobe of aedeagus and spermatheca as illustrated (Figs 7b, e)	***Mniusa odelli* Klimaszewski & Webster, sp. n.**
–	Body broadly subparallel, pronotum strongly transverse and basally slightly narrower than elytra (Fig. 6a); forebody moderately glossy, densely punctate and pubescent; elytra at suture longer than pronotum (Fig. 6a); median lobe of aedeagus and spermatheca as illustrated (Figs 6b, e)	***Mniusa yukonensis* (Klimaszewski & Godin)** [**BC**, YT, **QC**, **NB**, **NS**]

### 1. Mniusa minutissima

(Klimaszewski & Langor, 2011)

http://species-id.net/wiki/Mniusa_minutissima

Figure 5a–l , Map 3

Gnathusa minutissima Klimaszewski et al. 2011: 55.

#### Diagnosis.

Body length 2.0–2.3 mm, sides subparallel; body colour dark brown, with tarsi and often tibiae rust-brown, antennae brown; forebody with dense microsculpture, glossy and with moderately dense punctation and pubescence; head round, narrower than pronotum; pronotum transverse, rectangular in shape with sides feebly arcuate, and as wide as elytra; elytra at suture as long as pronotum (Fig. 5a); abdomen subparallel, narrower than elytra with deep basal impressions on first three visible tergites; antennae with articles V-X strongly transverse, with the outer segments at least twice as wide as long (Fig. 5a). MALE: male tergite VIII widely truncate apically (Fig. 5c); sternite VIII slightly produced at apex (Fig. 5d); median lobe of aedeagus with straight venter of tubus slightly arched laterally and internal sac with band-formed, subapical structure (Fig. 5b). FEMALE: tergite VIII truncate apically (Fig. 5f); sternite VIII rounded apically (Fig. 5g); spermatheca with small spherical capsule with small invagination, short L-shaped neck, and long, thin and broadly curved stem (Fig. 5e).

#### Distribution.

This native Nearctic species was described from Newfoundland and is herein recorded for the first time from New Brunswick (Map 3).

#### Bionomics.

Adults were collected from May to July using pitfall traps in an old boreal balsam fir forest in Newfoundland; by sifting moss near a brook, sifting deep conifer litter at base of large red spruce in a mature red spruce forest, and from Lindgren funnel traps in a rich Appalachian hardwood forest in New Brunswick.

#### Locality data.

CANADA: **Newfoundland:** Little Grand L., 2 km E. Martin Pond, 24.VI–15.VII.1992, old fir forest, pitfall 13, (LFC) 1 male [holotype]; same data except: pitfall 19, (CFS-CB) 1 male and 1 female [paratypes]; same data except: pitfall 20, 2 males and 1 female [paratypes]; pitfall 16, 1 female [paratype]; pitfall 13, 1 female [paratype]; pitfall 20, (LFC) 1 female paratype; Little Grand L., Bakeapple Brook, 24.VI–15.VII.1992, old fir forest, pitfall 1, (CFS-CB) 1 male [paratype]; same data except: pitfall 4, 1 male; pitfall 11, 1 female [paratype]; pitfall 3, (LFC) 1 female [paratype]; Manuals R., 8 km W. St. John’s, 10.VI.1984, D. Langor, Lot, (CFS-CB) 1 female [paratype]. **New Brunswick:** Sunbury Co., Acadia Research Forest, 45.9799°N, 66.3394°W, 14.V.2007, 18.VI.2007 // mature red spruce and red maple forest, sifting moss near brook, R.P. Webster (LFC, RWC) 3 males, 2 females; same locality data and forest type but 14.V.2007 // sifting deep conifer litter at base of large red spruce (RWC) 2 females: Carleton Co., Jackson Falls, “Bell Forest”, 46.2200°N, 67.7231°W, 4–12.VI.2008, R.P. Webster // Rich Appalachian Hardwood Forest with some conifers, Lindgren funnel trap (RWC) 1 female; same locality data and forest type but 1–8.VI.2009, 8–16.VI.2009, R. Webster & M.-A. Giguère, Lindgren funnel trap (RWC) 2 males.

### 2. Mniusa yukonensis

Klimaszewski & Godin

http://species-id.net/wiki/Mniusa_yukonensis

Figure 6a–l , Map 6

Ocyusa yukonensis Klimaszewski & Godin, in Klimaszewski et al. 2012: 218.

#### Diagnosis.

Body length 2.8–3.0 mm, narrowly elongate and broadest at elytra; body dark brown to almost black, sometimes with reddish tinge on elytra, reddish-brown antennae and legs; forebody with dense microsculpture, dense punctation and pubescence, and strongly glossy; head round and narrower than pronotum; pronotum transverse, with sides strongly arcuate and narrowed anteriad, slightly narrower than elytra; elytra at suture about as long as pronotum or slightly longer (Fig. 6a); abdomen broadly arcuate laterally, slightly narrower than elytra and with basal impressions on first three visible tergites; antennal articles V-X slightly transverse (Fig. 6a). MALE: male tergite VIII truncate apically (Fig. 6c); sternite VIII rounded apically and slightly produced medially (Fig. 6d); median lobe of aedeagus with tubus straight and slightly produced ventrally and with complex structures of internal sac (Fig. 6b). FEMALE: tergite VIII truncate apically with slightly acute lateral angles (Fig. 6f); sternite VIII rounded apically and slightly produced apically (Fig. 6g); spermatheca with narrowly elongate sac-shaped capsule connected with L-shaped neck and narrow and long stem (Fig. 6e).

#### Distribution.

This native Nearctic species was recently described from the Yukon Territory (Klimaszewski et al. 2012), and is here newly reported from Nova Scotia, New Brunswick, Quebec, and British Columbia, which constitute new provincial records (Map 6).

**Maps 5–6.**
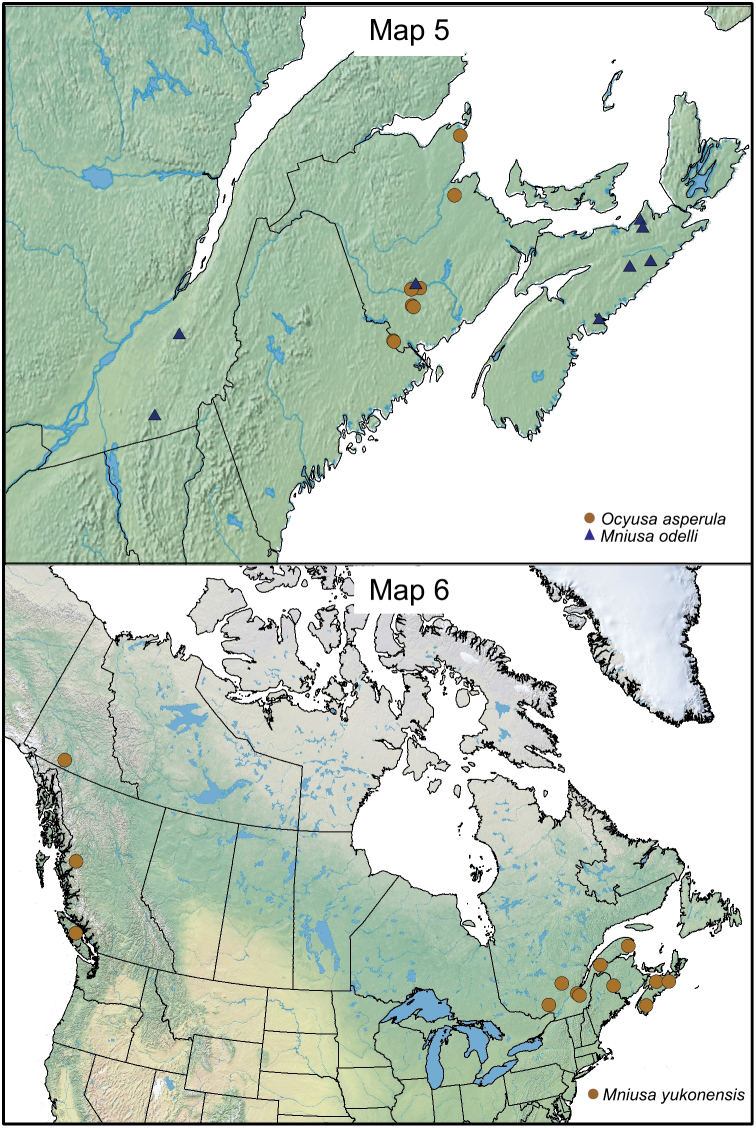


#### Bionomics.

Adults were collected from May to July in pitfall traps, flight intercept traps, and Lindgren funnel traps in various forest types: rich Appalachian hardwood forest with some conifers, old-growth white spruce/balsam fir forest, balsam fir and maple sugar stands, mature white spruce with feather moss, and a red spruce forest.

#### Locality data.

CANADA: **Nova Scotia:** Colchester Co., Debert, 6.V.1993, J. Ogden (NSPM) 1 male; Black Duck Lake, NS, 22.VI.2003, 4U: Funnel 16: 844 WPi/RSp (40-80 y), P. Dollin (NSPM) 1 male. **New Brunswick:** Carleton Co., Jackson Falls, “Bell Forest”, 46.2200°N, 67.7231°W, 4-12.VI.2008//, Rich Appalachian hardwood forest with some conifers, Lindgren funnel traps, R.P. Webster (RWC) 1 male, 2 females; same data except 12–19.VI.2008 (RWC) 1 female; same data except 19–27.VI.2008 (RWC) 1 female: same data except 26.V–1.VI.2009, R. Webster & M.-A. Giguère (RWC) 1 male; Restigouche Co., Dionne Brook P.N.A., 47.9064°N, 68.3441°W, 31.V–15.VI.2011 // old-growth white spruce and balsam fir forest, flight intercept trap, M. Roy & V. Webster (RWC) 1 male; same data except 31.V–1.VI.2011, Lindgren funnel trap (RWC) 3 males; same data except 15–27.VI.2011, Lindgren funnel trap (RWC) 1 female; same data except 21.VI–14.VII,2011, Lindgren funnel trap (RWC) 1 female. **Quebec:** Dosquet Co., Lotb. Quebec, 27.IV.1984, Claude Chantal (LFC) 1 male; Pelegrin, North of Chandler, 48°32'N, 64°54'W, SAP Lindgren, 21.06.1994 (LFC) 1 male; Tremblant, SAP Lindgren, 28.VI.1994 (LFC) 1 female; La Tuque, SAP Lindgren, 11.VII.1994 (LFC) 1 female; St-Jacques-de-Leeds, Sapinière Quebec, 31.V.1993, 9.VI.1993, 16.VI.1993, 28.VI.1993, 30.VI.1993, Lindgren 1, 2, 4 (LFC) 6 females. **British Columbia:** 25 km SW Campbell River, 49°50'21"N, 125°28'34"W, 23.V-6.VI.1996, Balsam Cr. LT 5-D 10, J. Lemieux (LFC) 4 males, 1 female, 1 sex undetermined; Copper River Valley, A36435/04-1-1, 07.VI-6.VII.1996, pitfall trap, J. Lemieux (LFC) 1 male. **Yukon Territory:** EMAN Plot (Ecological Monitoring and Assessment Network), mature white spruce and feather moss forest, 60.5963°N, 134.9522°W, 8.VII.2003, 738 m, yellow pitfall trap (LMKM31Y), (LFC) 1 male [holotype]; EMAN Plot, 60.5963°N, 134.9522°W, 24.VII.2003, 738 m, black pitfall trap (LMKM31B), (ECW) 1 male [paratype].

### 3. Mniusa odelli

Klimaszewski & Webster sp. n.

http://zoobank.org/49A0754A-850F-49B6-93C2-5F630C8A61CD

http://species-id.net/wiki/Mniusa_odelli

Figure 7a–l , Map 5

#### Holotype

**(female).** CANADA: **New Brunswick**, York Co., Fredericton, Odell Park, 45.9571°N, 66.6650°W, 15.V-1.VI.2012 // Old-growth eastern hemlock forest, Lindgren funnel trap, 1 m high under *Betula alleghaniensis*, C. Alderson and V. Webster (LFC). **PARATYPES:** New Brunswick, York Co., Odell Park, 45.9539°N, 66.6666°W, 10-24.VI.2013 // Hardwood stand, Lindgren funnel trap, 1 m high under trees (RWC) 2 females.

#### Diagnosis.

Body length 2.8–3.2 mm, narrowly subparallel with head slightly narrower than pronotum; body colour dark brown, and with tibiae, tarsi and often basal antennal articles reddish-brown; forebody with dense microsculpture, and moderately dense punctation and pubescence, and strongly glossy; head round and slightly narrower than pronotum; pronotum slightly transverse, with sides rounded, and as wide as elytra; elytra at suture about as long as pronotum or slightly longer (Fig. 7a); abdomen subparallel, as wide as elytra and with basal impressions on first three visible tergites; antennal articles V-X slightly transverse (Fig. 7a). MALE [description of male is based on poorly preserved specimen and should be consider as tentative]: male tergite VIII truncate apically (Fig. 7c); sternite VIII rounded apically and slightly produced medially (Fig. 7d); median lobe of aedeagus with tubus straight and with complex structures of internal sac (Fig. 7b). FEMALE: tergite VIII truncate apically with sharp lateral angles (Fig. 7f); sternite VIII rounded apically (Fig. 7g); spermatheca with narrowly elongate sac-shaped capsule connected with L-shaped neck and narrow and long stem slightly sinuate posteriorly at apex (Fig. 7e).

#### Distribution.

This native Nearctic species is here described from Nova Scotia, New Brunswick, and Quebec (Map 5).

#### Bionomics.

Adults were collected from May to July in Lindgren traps in an old-growth eastern hemlock stand, an old hardwood stand, and in a sugar maple forest and a red spruce forest.

#### Etymology.

This species is named after Odell Park in Fredericton, New Brunswick, where the holotype was found. This park was originally the estate of Reverend Jonathan Odell whom the park was named after. This park was established in 1954.

#### Other locality data

[specimens in poor condition and tentatively identified as this species].

CANADA: **Nova Scotia:** Pictou Co., Marshy Hope, 17.V.1995, ethanol lure, M. LeBlanc (NSPM) 1 sex undetermined; Antigonish Co., Fairmont Tower Road, 17.V.1995, 3-Component Lure w/+/- mcol, M. LeBlanc (NSPM) 1 female; Fairmont Tower Road, 25.V.1995, 3-Component Lure w/+/- mcol, M. LeBlanc (NSPM) 1 male, 1 female; Antigonish Co., Eigg Mountain, 25.V.1995, ethanol lure, M. LeBlanc (NSPM) 1 male, 1 female; Melopseketch Lake, Guy, 14.V–2.VI.1997, young red spruce, D.J. Bishop 201 (NSPM) 1 male; Halifax, Lake Little, 14.V–2.VI.1997, regenerating red spruce, D.J. Bishop 127 (NSPM) 1 male. **Quebec:** St-Jacques-de-Leeds, Erablière Québec, 28.VI.1993, Lindgren 2 (LFC) 1 female; Mont Orford, 29.VI-6.VII.1999, Lindgren 1, Erablière, 99-3-1061 (LFC) 1 male.

### Ocyusa

Kraatz

http://species-id.net/wiki/Ocyusa

Ocyusa Kraatz, 1856. Type species: *Oxypoda maura* Erichson.

#### Diagnosis.

**Figure 8.**
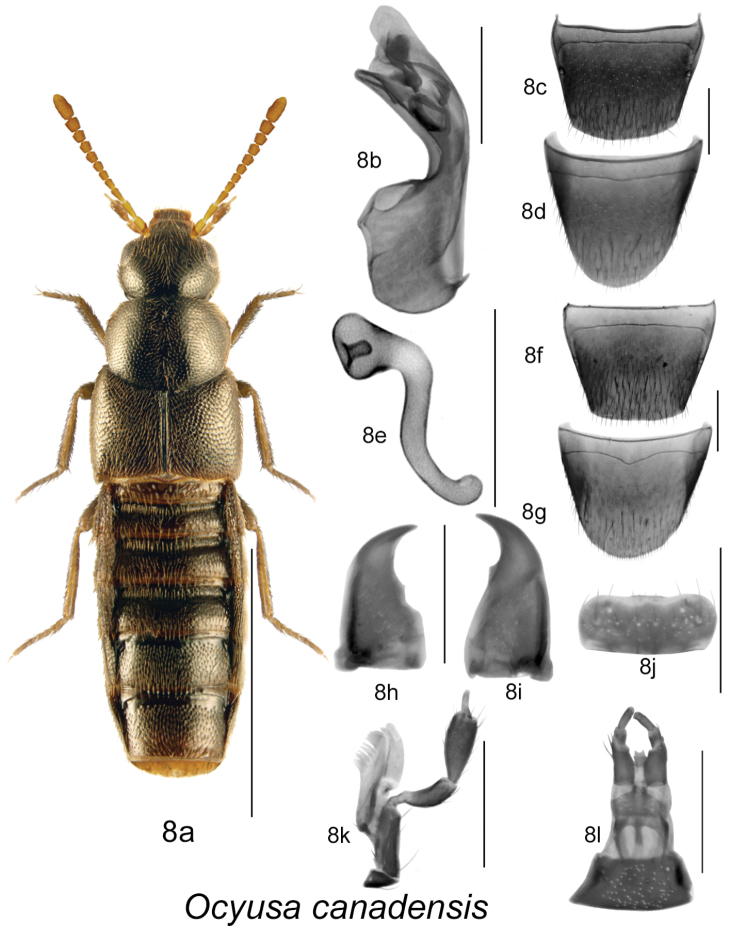
*Ocyusa canadensis* Lohse: **8a** habitus **8b** median lobe of aedeagus in lateral view **8c** male tergite VIII **8d** male sternite VIII **8e** spermatheca in lateral view **8f** female tergite VIII **8g** female sternite VIII **8h** left mandible **8i** right mandible **8j** labrum **8k** maxilla **8l** menthum, labial palpi and ligula. Habitus scale bar = 1.0 mm; other scale bars = 0.2 mm.

**Figure 9.**
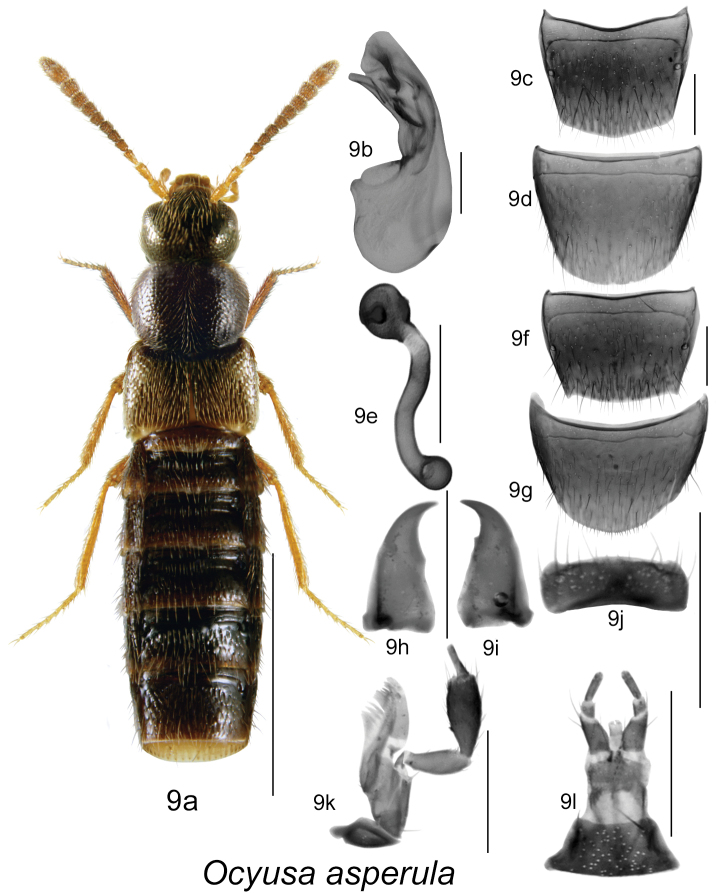
*Ocyusa asperula* Casey: **9a** habitus **9b** median lobe of aedeagus in lateral view **9c** male tergite VIII **9d** male sternite VIII **9e** spermatheca in lateral view **9f** female tergite VIII **9g** female sternite VIII **9h** left mandible **9i** right mandible **9j** labrum **9k** maxilla **9l** menthum, labial palpi and ligula. Habitus scale bar = 1.0 mm; other scale bars = 0.2 mm.

Body dark brown to almost black, compact, sides subparallel or body narrowly oval in outline (Figs 8a, 9a), length 2.5–3.5 mm; integument with distinct meshed microsculpture and moderate to dense punctation and pubescence; head large with mandibles broad and long, left mandible with a small tooth (Figs 8h, 9h), and right one with a slightly larger tooth at the base of arcuate cutting edge of mandible, apices moderately narrowly elongate [less than in *Mniusa*] (Figs 8i, 9i); infraorbital carina strong and complete; ligula shallowly split apically (Figs 8l, 9l); labial palpus with three articles, second article minute, last one needle-shaped and lacinia and galea as illustrated (Figs 8k, 9k); labrum narrow and transverse, apical edge entire (Figs 8j, 9j); frontal suture of head present; pronotal pubescence along midline directed posteriad or obliquely posteriad; anterior margin of mesosternum without longitudinal carina; mesosternal process triangular basally and then produced and extending to about 2/3 length of mesosternal cavities, metasternal process narrowly triangular in shape and short; isthmus short; median lobe of aedeagus strongly produced ventrally, internal sac structures as illustrated (Figs 8b, 9b); spermatheca S-shaped with spherical capsule, short neck and elongate sinuate stem (Figs 8e, 9e).

#### Key to Canadian species of *Ocyusa*

**Table:** 

1	Elytra at suture shorter than pronotum (Fig. 9a); forebody strongly glossy, sparsely punctate and pubescent; median lobe of aedeagus and spermatheca as illustrated (Figs 9b, e)	***Ocyusa asperula* Casey** [NB, RI, MA, IA]
–	Elytra at suture about as long as pronotum (Fig. 8a); forebody moderately glossy, densely punctate and pubescent; median lobe of aedeagus and spermatheca as illustrated (Fig. 8b, e)	***Ocyusa canadensis* Lohse** [AK, YT, ON, NB, NF]

### 1. Ocyusa canadensis

Lohse

http://species-id.net/wiki/Ocyusa_canadensis

Figure 8a–l , Map 4

Ocyusa canadensis Lohse, in Lohse et al. 1990: 147; Brunke et al. 2012: 134.

#### Diagnosis.

Body length 2.5–3.0 mm, narrowly elongate and broadest at elytra; body colour dark brown to almost black, with tarsi, two basal antennal articles and tibiae rust-brown, rest of antennal articles brown; forebody with dense microsculpture, moderate punctation and pubescence, and strongly glossy; head round and narrower than pronotum; pronotum transverse, rectangular in shape with sides strongly arcuate, and narrower than elytra; elytra at suture about as long as pronotum (Fig. 8a); abdomen broadly arcuate laterally, slightly narrower than elytra and with basal impressions on first three visible tergites; antennae with articles V-X subquadrate to slightly elongate (Fig. 8a). MALE: male tergite VIII broadly rounded apically (Fig. 8c); sternite VIII rounded apically (Fig. 8d); median lobe of aedeagus with tubus strongly bent ventrally and with pronounced structures of internal sac (Fig. 8b). FEMALE: tergite VIII truncate apically (Fig. 8f); sternite VIII rounded apically (Fig. 8g); spermatheca with semi-spherical capsule with long invagination, and S-shaped broad stem (Fig. 8e).

#### Distribution.

This native Nearctic species is known from Alaska, Yukon Territory and Ontario (Brunke et al. 2012), and is here newly reported from the island of Newfoundland and New Brunswick [new provincial records] (Map 4).

#### Bionomics.

Some adults were collected from June to July at lake margins, on moist soil/gravel among sedges and by treading *Carex* and grasses.

#### Locality data.

CANADA: **Newfoundland:** George’s Lake, Corner Brook, 29.VII.1972, J.M. Campbell (CNC) 1 male; **New Brunswick:** Restigouche Co., Wild Goose Lake, 419 m elevation, 47.8540°N, 68.3200°W, 21.VII.2010 // lake margin, on moist soil/gravel among sedges, R.P. Webster (RWC) 1 female; same data except: 420 m elevation, 47.8543°N, 68.3219°W, 7.VI.2011 // lake margin with emergent *Carex* and grasses, treading *Carex* and grasses, R.P. Webster (RWC) 1 male; same data except 20.VI.2011, (RWC) 2 males, 4 females. **Ontario:** Timiskaming Distr., 52 km South Armstrong, 27.VI.1973, R. Parry and J.M. Campbell (CNC) 4 females, 7 sex undetermined; 52 km S Armstrong, 27.VI.1973, R. Parry and J.M. Campbell (CNC) 1 female. **Yukon Territory:** Dempster Hwy., mi. 122, 20.VII.1978, 2000 feet, A. Smetana and J.M. Campbell (CNC) 2 males, holotype and paratype; Dempster Hwy., mi. 147, 1900 feet, 22.VII.1978, J.M. Campbell and A. Smetana (CNC) 1 sex undetermined.

UNITED STATES OF AMERICA: **Alaska:** Prudhoe Bay Rd., Bonanza Creek, 900 feet, 66°40'N, 150°40'W, 2.VII.1978, A. Smetana and J.M. Campbell (CNC) 1 paratype sex undetermined; Nutirwick Creek, 67°55'N, 149°45'W, 2300 feet, 8.VII.1978, J.M. Campbell and A. Smetana (CNC) 2 sex undetermined.

### 2. Ocyusa asperula

Casey

http://species-id.net/wiki/Ocyusa_asperula

Figure 9a–l , Map 5

Ocyusa asperula Casey 1894: 305 [often cited as 1893], Webster et al. 2009: 192.
Ocyusa brevipennis Bernhauer 1906: 344. Moore and Legner 1975: 458. Synonymy confirmed.

#### Diagnosis.

Body length 2.8–3.0 mm, sides subparallel; body colour dark brown, with tarsi, two basal antennal articles and legs rust-brown, rest of antennal articles dark brown; forebody with moderately dense microsculpture, punctation and pubescence, and strongly glossy; head round and about as wide as pronotum; pronotum transverse, with sides strongly arcuate, widest in apical third, and as wide as elytra; elytra at suture much shorter than pronotum (Fig. 9a); abdomen broadly arcuate laterally, slightly broader than elytra at middle and with basal impressions on first three visible tergites; antennae with articles V-X subquadrate (Fig. 9a). MALE: male tergite VIII with apical margin slightly pointed medially (Fig. 9c); sternite VIII rounded apically (Fig. 9d); median lobe of aedeagus with tubus convex basally and then strongly bent ventrally and with complex structures of the internal sac (Fig. 9b). FEMALE: tergite VIII truncate apically with small projections laterally (Fig. 9f); sternite VIII rounded apically (Fig. 9g); spermatheca with small spherical capsule with long and broad invagination, and S-shaped broad stem slightly swollen posteriorly (Fig. 9e).

#### Distribution.

This native Nearctic species was described from Rhode Island by Casey 1894 [often cited as 1893]. It was recorded also from New Brunswick in Canada (Map 5) and from Iowa and Massachusetts in the United States (Casey 1894, Bernhauer 1906 [as *brevipennis*], Moore and Legner 1975, Webster et al. 2009).

#### Bionomics.

Some adults were collected from April to July at lake margins, on moist soil/gravel among sedges, and by treading emergent *Carex* and grasses. Webster et al. (2009) collected adults by sifting grass litter and mosses (usually sphagnum) near small pools in eastern white-cedar swamps, red maple (*Acer rubrum* L.) swamps with eastern white-cedar, and in alder swamps. Others were collected by treading green sphagnum, *Carex*, and grasses in a black spruce bog and by treading cattails and sedges in a boggy marsh.

#### Locality data.

CANADA: **New Brunswick:** Charlotte Co., 3 km SW of King Brook Lake, 45.3194°N, 67.4414°W, 27.V.2007 (RWC) 1 sex undetermined; 3.0 km NW of Pomeroy Ridge, 45.3059°N, 67.4343°W, 5.VI.2008 (RWC) 1 sex undetermined; Gloucester Co., ca. 1.5 km NE of Six Roads, off Paleot Rd., 47.6292°N, 64.8565°W, 32.V.2010, R.P. Webster (RWC) 1 sex undetermined; Northumberland Co., Goodfellow Brook PNA, 46.8943°N, 65.3796°W, 23.V.2007 (BM) 1 sex undetermined; York Co., New Maryland, off Hwy 2, E of Baker Brook, 45.8760°N, 66.6252°W, 6.IV.2005 (RWC) 1 male; near Mazerolle Settlement, 45.8987°N, 66.7903°W, 9.IV.2006, R.P. Webster (LFC, RWC, NBM) 6 males, 3 females; 9.2 km W of Tracy off Rt. 645, 45.6837°N, 66.8809°W, 22.V.2008 (RWC) 1 female; ca. 14 km SW of Tracy, S of Rt. 645, 45.6603°N, 66.8603°W, 2.VII.2010, R.P. Webster (RWC) 1 sex undetermined.

## Supplementary Material

XML Treatment for Gnathusa

XML Treatment for Gnathusa eva

XML Treatment for Gnathusa tenuicornis

XML Treatment for Gnathusa caribou

XML Treatment for Gnathusa alfacaribou

XML Treatment for Mniusa

XML Treatment for Mniusa minutissima

XML Treatment for Mniusa yukonensis

XML Treatment for Mniusa odelli

XML Treatment for Ocyusa

XML Treatment for Ocyusa canadensis

XML Treatment for Ocyusa asperula
